# Drugs and Epigenetic Molecular Functions. A Pharmacological Data Scientometric Analysis

**DOI:** 10.3390/ijms22147250

**Published:** 2021-07-06

**Authors:** Dario Kringel, Sebastian Malkusch, Jörn Lötsch

**Affiliations:** 1 Institute of Clinical Pharmacology, Goethe-University, Theodor Stern Kai 7, 60590 Frankfurt am Main, Germany; kringel@med.uni-frankfurt.de (D.K.); malkusch@med.uni-frankfurt.de (S.M.); 2 Fraunhofer Institute for Translational Medicine and Pharmacology ITMP, Theodor-Stern-Kai 7, 60596 Frankfurt am Main, Germany

**Keywords:** pharmacological data science, pharmacoepigenetics, computational knowledge-discovery, pharmacological plasticity

## Abstract

Interactions of drugs with the classical epigenetic mechanism of DNA methylation or histone modification are increasingly being elucidated mechanistically and used to develop novel classes of epigenetic therapeutics. A data science approach is used to synthesize current knowledge on the pharmacological implications of epigenetic regulation of gene expression. Computer-aided knowledge discovery for epigenetic implications of current approved or investigational drugs was performed by querying information from multiple publicly available gold-standard sources to (i) identify enzymes involved in classical epigenetic processes, (ii) screen original biomedical scientific publications including bibliometric analyses, (iii) identify drugs that interact with epigenetic enzymes, including their additional non-epigenetic targets, and (iv) analyze computational functional genomics of drugs with epigenetic interactions. PubMed database search yielded 3051 hits on epigenetics and drugs, starting in 1992 and peaking in 2016. Annual citations increased to a plateau in 2000 and show a downward trend since 2008. Approved and investigational drugs in the DrugBank database included 122 compounds that interacted with 68 unique epigenetic enzymes. Additional molecular functions modulated by these drugs included other enzyme interactions, whereas modulation of ion channels or G-protein-coupled receptors were underrepresented. Epigenetic interactions included (i) drug-induced modulation of DNA methylation, (ii) drug-induced modulation of histone conformations, and (iii) epigenetic modulation of drug effects by interference with pharmacokinetics or pharmacodynamics. Interactions of epigenetic molecular functions and drugs are mutual. Recent research activities on the discovery and development of novel epigenetic therapeutics have passed successfully, whereas epigenetic effects of non-epigenetic drugs or epigenetically induced changes in the targets of common drugs have not yet received the necessary systematic attention in the context of pharmacological plasticity.

## 1. Introduction

Epigenetics summarizes mechanisms of DNA transcription regulation and therefore plays a ubiquitous role in biological processes. It deals with the regulation of DNA transcription in the absence of changes in the DNA sequence [1]. Classical epigenetics involves the regulation of DNA transcription at two main sites, namely (i) at the DNA itself by adding or removing methyl groups to or from cytosines, thereby facilitating or inhibiting transcription, and (ii) at histones, which are proteins around which DNA wraps and thus regulate the conformation of DNA as a component of chromatin, by modifying the exposure of the DNA to transcription factors. Their involvement in most biological regulatory pathways from the starting points and at all subsequent steps via regulation of the expression of the proteins involved makes epigenetic mechanisms potential therapeutic targets for a wide range of pathological processes [2].

Epigenetic changes can be triggered by various factors. It is known that changes in DNA methylation are primarily caused by chemicals such as medicinally used drugs [3], royal jelly [4] and various toxins such as benzene [5], asbestos or smoking [6], or food [7]. Non-chemical factors include lifestyle or experiences such as physical activity [8], maternal care [9,10], psychological trauma [11] or chronic pain [12]. Changes in histone modifications have been associated with genome instability, chromosome segregation defects, and cancer [13]. For example, homozygous null mutant embryos for the *SET7* gene show early lethality due to defective mitotic chromosome condensation [14]. In addition, histone modifications can be induced by various factors such as drug, nicotine or alcohol abuse [15,16,17,18].

The coordinated actions of proteins involved in the epigenetic regulation of DNA transcription determine cell development, cell cycle regulation, cell state and fate, and final responses in health and disease. Therefore, they have been identified as targets of a new class of epigenetic drugs developed mainly for novel treatments of various cancers [2]. However, pharmacological research of pleiotropic effects of common drugs has identified epigenetic mechanisms as effects of various non-epigenetic drugs [3], i.e., drugs that were not designed with epigenetic pathways in mind, such as agonists of G-protein coupled receptors [19,20], cyclooxygenase inhibitors [21], or ion channel blockers [22].

Therefore, the present scientometric original report assesses known interactions of current drugs, including drugs that are not primarily epigenetic, with targets involved in epigenetic regulation that have been explored using computational methods of knowledge discovery in publicly available databases (Table 1). This has taken advantage of the increasing availability of computational methods in biomedical research that allow current knowledge about the biological roles of genes and their products and drug-drug interactions with target proteins to be stored in databases and this knowledge to be retrieved and combined in an automated manner. This enables the use of acquired knowledge about genes, epigenetic targets over drugs for mapping the molecular mechanisms addressed by drugs with the incorporation of computational or systems biology methods in pharmacological research.

## 2. Results

### Enzymes Involved in Epigenetic Processes

A total of *n* = 275 unique enzymes involved in classical epigenetic processes were queried from the HEMD database. All players of DNA modifications (methylation and demethylation, e.g., *n* = 5 and 14 enzymes, respectively) and all enzymes catalyzing histone conformational changes were included. The identified enzymes provided the systematic basis for further analyses of the current state of knowledge on the interactions between drugs and classical epigenetic processes represented by their genetic targets. The genes encoding the enzymes for which drug interactions were identified in later stages of data analysis are listed in Table 2.

## 3. Literature-Search Based Evidence for Epigenetic Drug Effects

### 3.1. Bibliometric Characteristics of Publication Activities on Epigenetic Drug Effects

**Figure 1:**
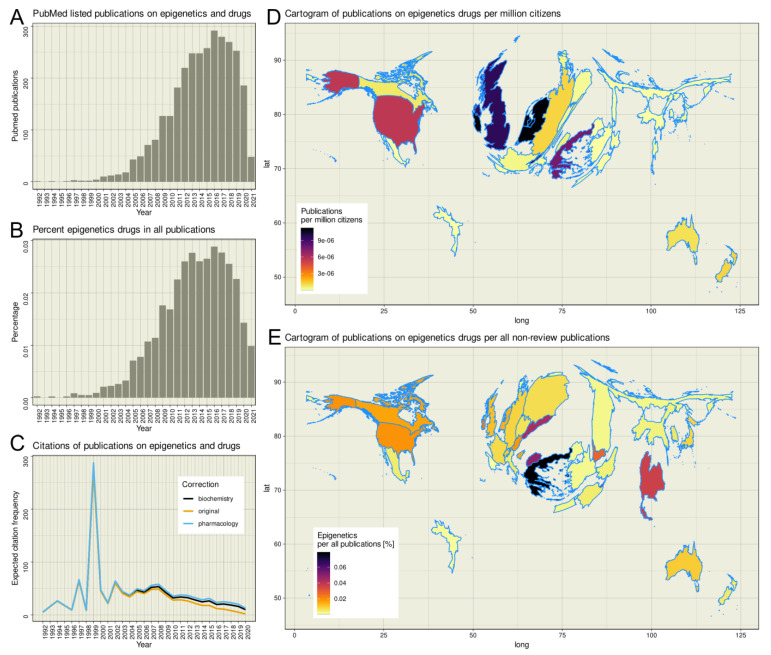
Bibliometric exploration of PubMed listed publications on the topic of epigenetic drug effects. Results of a computed PubMed database analysis of year, citation count, and country of origin of publications on epigenetics and drugs not listed as reviews. (**A**) Bar chart of the number of publications per year, starting with the first publication on epigenetics and drugs in 1992. (**B**) Bar chart of the respective annual percentage of publications on epigenetics and drugs out of all publications listed in PubMed that were not of publication type “review”. (**C**) Line graph of citations of publications identified in panel A. The observed or expected average cumulative number of citations per article measured over the period from publication date to query ${\bar{n}}_{cit,\Delta t}$ is plotted against the publication year (dark-yellow line). T$\langle {\bar{N}}_{cit}\rangle$ per publication is calculated considering the citation behavior of a readership either from the field of biochemistry and molecular biology ($\beta_{bio}=5.4$, black line) or pharmacology ($\beta_{pharma}=7.1$, blue line). The parameter β denotes the time period in which a scientific article achieves 63% of its total reference and thus, accounts for citation cultures in different scientific disciplines or topics. (**D**) Publication activity per country standardized at the average population of the respective country during the analyzed period, plotted as spatial plots with Gaussian blur as described in [35], with boundaries of regions transformed to be proportional to publication counts. Publications were summed for the period 1992 (first publication on epigenetics and drugs) to 2020 (last entry in the United Nations World Population Report). (**E**) Mapping of publications on epigenetics and drugs per country normalized to all publications listed in PubMed between 1992 and 2021 for the respective country. The figures has been created using the software package R (v4.0.5 for Linux; https://CRAN.R-project.org/ (R Development Core Team, 2008)) and the libraries “ggplot2” (https://cran.r-project.org/package=ggplot2 [36]) and “Rcartogram” (https://github.com/omegahat/Rcartogram [37]).

The R-based PubMed database search yielded 3051 hits; using an empty search string yielded 32,182,784 hits, of which 2,794,483 were reviews. Thus, epigenetic drug effects account for only up to 0.0288% of all non-review publications listed in PubMed, with a maximum of 292 hits for the year 2016 (Figure 1). The earliest publication on epigenetics and drugs was a report of the effects of 5-azacytidine on DNA methylation [31] dated 1992. Since then, the citations per year increased until a plateau is reached in 2000 (Figure 1C). In the first decade of the new millennium, the annual citation rate of publications dealing with epigenetics remained constant until 2008, when a steady downward trend in the citation rate began, which continues to this day. Applying corrections for the shorter time since publication according to citation habits in the scientific communities of biochemistry or pharmacology [32] resulted in more citations, especially of recent work, as expected citations were additionally considered; however, the negative trend was not broken. The most cited publication is dated 2012 [33]. The publication reports on the potential of direct GSK126-mediated inhibition of EZH2 methyltransferase activity as an inhibitor for the growth of EZH2 mutant diffuse large B-cell lymphoma xenografts in mice. At the time the literature search was done it has been cited 702 times in total. The second most cited publication is dated 1999 [34]. It describes the fact that the histone deacetylase inhibitor trichostatin A upregulates the expression of non-methylated genes in tumor cells, while it is unable to reactivate hypermethylated genes. At the time of the literature search it has been cited 528 times. The peak of the publication curve at 1999 is due to the low number of publications in the field at that time, coupled with the fact that one of the two publications is one of the two most cited publications in the research field. The influence of exceptionally frequently cited publications on the annual average of references per publication only becomes relative as the statistics increase. This can be seen in the publication curve around 2012. Although the most cited paper [33] of the subject area was published in this year, its 702 references could not stop the negative trend of the subject area, which started at this time.

Analysis of worldwide research activity revealed that publications on epigenetics and drugs originated from 34 countries, with the highest contribution from the United States (*n* = 1676 hits), followed by the United Kingdom (*n* = 594) and the Netherlands (*n* = 183). When the number of publications per year was normalized to the respective country population, averaged over the years in which the publications were dated, the weight shifts toward Western Europe, with Ireland, the Netherlands, Switzerland, and the United Kingdom having the largest proportion of publications per capita, followed by Greece (Figure 1D). Taking the total number of non-review publications over the same period as a reference, it appeared as if the topic was particularly strong in biomedical research in Greece and Northern Macedonia (Figure 1E).

### 3.2. Published Evidence of Epigenetic Drug Effects

The PubMed database search using the above-mentioned search string yielded three main categories of results on epigenetics and drugs: (i) drugs targeting DNA modifications, (ii) drugs targeting histone modifications, and (iii) evidence of distinct epigenetic modulations of the responses to drugs through regulatory interventions in the expression of the respective targets. Drugs targeting epigenetic mechanisms comprised substances that had been developed with the epigenetic target in the focus (Table 3). However, there is also evidence that drugs with other main targets may nevertheless affect epigenetic mechanisms as a pleiotropic effect, which provided important or additional explanations for their clinical effects that are not covered by their main, non-epigenetic targets (Table 4). The three categories mentioned above are exemplarily described in the following sections.

### 3.3. Drugs Targeting DNA Methylation

Of all epigenetic modifications, methylation and acetylation are the best studied. In DNA methylation, cytosine nucleotides are methylated by various DNA methyltransferases (DNMTs), especially at so-called CpG motifs or CpG islands. During cell division, these DNA modifications are transferred to the daughter cells. Methylation leads to “silencing” of the corresponding DNA segment. There is evidence that this plays a role in carcinogenesis. For example, tumor suppressor genes and genes responsible for DNA repair have been found to have hypermethylated sections in the promoter region that interfere with the reading of DNA information [76]. Therefore, in the early 2000s, inhibition of DNA methylation was recognized as a promising target for antitumor agents, leading to the approval of the DNA methyltransferase inhibitors azacytidine and decitabine for the treatment of myelodysplastic syndromes in the United States. These nucleoside inhibitors of DNA methylation have become standard of care also in the treatment of the myelodysplastic syndrome, a fatal form of leukemia [77].

### 3.4. Drugs Targeting Histone Conformation

The major clinical application of inhibitors of histone deacetylases (HDACs) is again in oncology, due to their modulatory effects on cell cycle and gene expression [78]. Possibilities for modification of histones include reversible lysine acetylation, since histones are rich in lysine and arginine, by HDACs and histone acetyltransferases (HATs), and phosphorylation of serine or threonine. In 2006, the first histone deacetylase inhibitor, vorinostat, received U.S. approval for the treatment of advanced cutaneous T-cell lymphoma [79]. Inhibition of HDAC by vorinostat leads to hyperacetylation of histones and causes, among other things, stagnation in the cell cycle to trigger apoptotic processes, inhibit angiogenesis and leads to the destruction of tumor cells [80]. Panobinostat is also being tested in clinical trials for the treatment of myelodysplastic syndromes and was approved for medical treatment in 2015 [81]. It is an antiproliferative and cytotoxic agent from the group of HDAC inhibitors used to treat multiple myeloma [82]. The effect is based on the inhibition of histone deacetylases, which leads to an accumulation of acetylated histones and ultimately to cell death. The HDAC inhibitor belinostat for the treatment of peripheral T-cell lymphoma has also successfully passed clinical trials (25802282), as the long-used antiepileptic agent valproic acid, which is also capable of inhibiting HDAC activity and is accessible to the central nervous system, so that trials have been initiated for the treatment of brain tumors [83]. However, because the HDAC inhibitory effect of valproic acid is relatively weak, combined use with other agents, such as lenalidomide, is endorsed [84].

### 3.5. Epigenetic Modulation of Drug Responses

Tamoxifen is used in both premenopausal and postmenopausal women as an adjuvant treatment for hormone receptor-positive breast cancer. An important obstacle to its use is the development of drug resistance caused by molecular processes related to genetic mechanisms, such as the action of cytochrome P450 2D6 (CYP2D6) polymorphisms which influence the activation of tamoxifen to enoxifen [85]. CYP2D6 is also subject to epigenetic modulation at the level of DNA methylation and histone methylation [86]. Thus, if pharmacogenetic regulation of its effects has been described, it is conceivable that tamoxifen is also subject to pharmacoepigenetic regulation via epigenetic regulation of its CYP2D6-mediated activation to enoxifen. This is similarly true for codeine, which must be demethylated to morphine [87] and for other CYP2D6 substrates [88]. Similarly, other drug metabolizing enzymes and transmembrane transporters that have drugs among their subject are subject to epigenetic regulation that has been reviewed elsewhere [89].

An example of clinically relevant epigenetic regulation of a drug target is the reduced expression of µ-opioid receptors in an *OPRM1* A > G genetic variant (dbSNP database [90] accession number rs1799971) that introduces an additional CpG methylation site into a transcription-relevant DNA region [91]. Other examples include epigenetic modulation of glucocorticoid receptors in patients with post-traumatic stress disorder (PTSD), where the transmission to the offspring was explained by the transmission of epigenetic processes such as the methylation status of the glucocorticoid receptor gene *NR3C1* [92,93]. Of note, the transmission of hypermethylation has been followed from cross-sectional observations in parents and children. A recent study showed that human glucocorticoid receptor expression was increased in peripheral blood samples from individuals with lifelong PTSD. These differences in expression were associated with general and site-specific DNA hypomethylation, suggesting that traumatic events in PTSD induce DNA methylation changes that modify gene expression and HPA axis activity, a well-characterized feature in PTSD [94]. In a B-lymphoblastic leukemia cell lines, increase of HDAC3 levels in the glucocorticoid signaling pathway resulted in resistance to glucocorticoid agonists [95].

## 4. Automated Analysis of Interactions of Drugs with Epigenetic Targets

A query of the DrugBank database in April 2021 identified 14,315 drugs. The substances were grouped into approved (*n* = 4108), experimental (*n* = 6554), illicit (*n* = 205), investigational (*n* = 5245), nutraceutical (*n* = 131), veterinary approved (*n* = 423) and withdrawn (*n* = 265); the higher sum of group members over the total number of drugs is due to the assignment of some drugs to more than one group. The drugs interacted with a total of *n* = 4885 unique targets, of which 2914 were human proteins.

**Figure 2:**
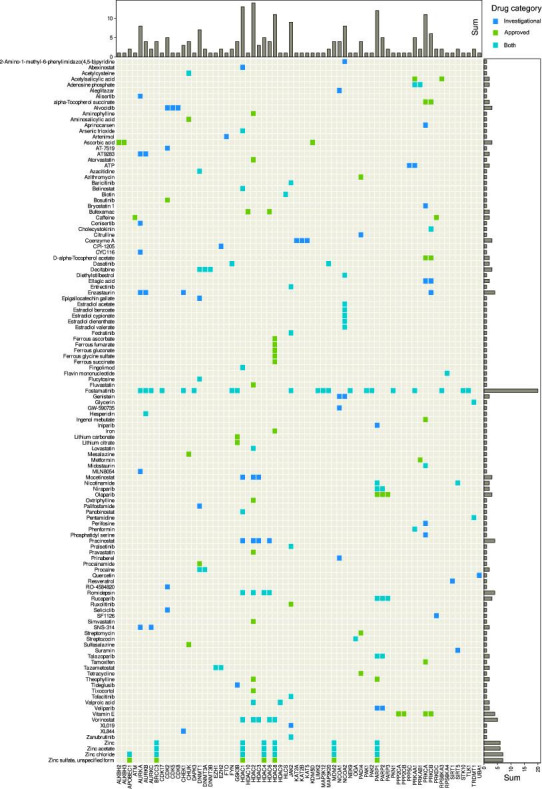
Interactions of approved and/or investigational drugs, queried from the DrugBank database [24], with epigenetic enzymes, queried from the HEMD database [26]. Matrix heat plot of mutual interactions of dugs with genes coding for enzymes involved in epigenetic regulation (Table 2). The marginal statistics are presented as bar plots indicating row or column sums of interactions per drug or target, respectively. The figure has been created using the R software package (v4.0.5 for Linux; https://CRAN.R-project.org/ [29]), and the R library “ComplexHeatmap” (https://bioconductor.org/packages/release/bioc/html/ComplexHeatmap.html [98]).

Of the 275 queried enzymes from the HEMD database, *n* = 82 were annotated with a total of 401 drugs. Among annotated drugs, *n* = 82 were approved drugs and *n* = 85 were investigational drugs. Of the remaining drugs *n* = 283 were experimental drugs, which often included chemical names of compounds, such as “3-[3-(2,3-Dihydroxy-Propylamino)-Phenyl]-4-(5-Fluoro-1-Methyl-1h-Indol-3-Yl)-Pyrrole-2,5-Dione”, for which clinical utility cannot yet be predicted, *n* = 10 were nutraceutical substances, *n* = 12 veterinary approved, and *n* = 4 were withdrawn. Only approved and investigational drugs were considered further to limit the focus of this report to epigenetic drug effects with likely therapeutic relevance. These drug groups contained *n* = 122 unique drugs, owing to the dual group assignment of some substances. They interacted with *n* = 68 unique epigenetic enzymes (Table 2). Between approved or investigational unique drugs and epigenetic enzymatic targets, a total of 213 interactions was found (Figure 2).

The most interactions with enzymes involved in epigenetic processes had fostamatinib, an inhibitor of the spleen-associated tyrosine kinase encoded by the *SYK* gene (NCBI accession number 6850). The drug is approved for the treatment of refractory immune thrombocytopenia [96] and has also been shown to be an effective and safe therapeutic for rheumatoid arthritis [97]. It is listed in DrugBank with 300 different targets, 20 of which are among the here analyzed genes that encode epigenetic enzymes (*AURKA*, *AURKB*, *AURKC*, *CDK17*, *CHEK1*, *DAPK3*, *FYN*, *GSK3B*, *JAK2*, *LIMK2*, *MAP3K12*, *MAP3K20*, *NEK9*, *PAK1*, *PAK2*, *PKN1*, *PRKAA1*, *RPS6KA3*, *STK10*, *TLK1*). They encode enzymes involved in histone modification, while DNA methylation seems to be unaffected by fostamatinib.

Among enzymes with functions in epigenetic processes, histone deacetylase 2 was most frequently modulated by a drug, according to the DrugBank records (Figure 2). It is the target of drugs specifically designed for its epigenetic activity, such as vorinostat, which has the chemical name *N*-hydroxy-*N*’-phenyloctanediamide and is approved for histone deacetylase inhibition in the treatment of advanced, refractory, cutaneous T-cell lymphoma [99]. In addition to HDAC2, vorinostat interacts with HDAC1, HDAC3, HDAC6 and HDAC8 (Figure 2). The drug is also active against other cancers such as prostate cancers with which it was first published [100] and several further malignancies [101]. It is also being contemplated as a component of an HIV cure [102] that goes beyond the current therapeutic success of suppressing the virus without eliminating it from the patient’s body, or as a potential antiparasitic [103].

However, primarily non-epigenetic drugs designed with other targets in mind are also targeting HDAC2, for example, valproic acid, a nearly 140-year-old drug [104] used in the therapy of epilepsy due to blockade of voltage-gated sodium channels and GABAergic effects [105,106]. The drug has been repurposed for persistent pain, particularly lancinating neuropathic pain [107,108] including diabetic neuropathy [109]. While its main mechanism of action contributes to this successful expansion of its therapeutic spectrum, it has also been recognized that pain modulation is mediated via inhibition of histone acetylation [110], which can be attributed to the interaction of valproic acid with HDAC2 [111]. It also interacts with HDAC9, which has been proposed as a therapeutic approach in the preventive treatment of ischemic stroke [112].

**Figure 3:**
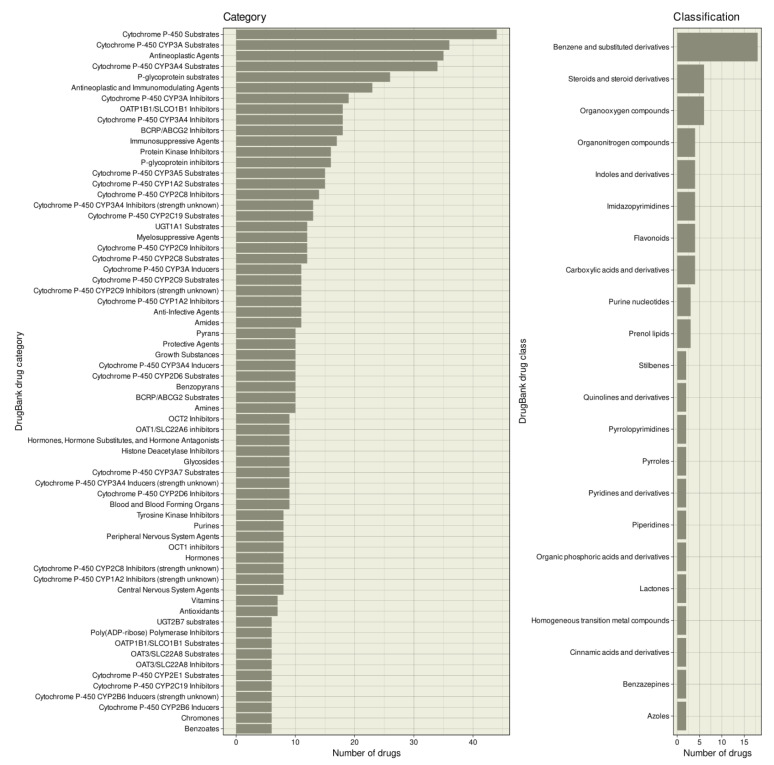
Categories and classes to which epigenetic drugs have been assigned. Bar plot of DrugBank categories and drug classes to which the drugs with epigenetic effects (Figure 2) are assigned in the DrugBank database. The figure has been created using the R software package (v4.0.5 for Linux; https://CRAN.R-project.org/ [29]) and the R library “ggplot2” (https://cran.r-project.org/package=ggplot2 [36]).

The drugs interacting with enzymes involved in epigenetic processes belonged to 646 different categories of the DrugBank nomenclature, of which categories assigned to at least five drugs are shown in Figure 3. Apart from the fact that most drugs were “antineoplastic agents” (*n* = 35), “antineoplastic and immunomodulatory agents” (*n* = 23), “immunosuppressive agents” (*n* = 17), or “myelosuppressive agents” (*n* = 12), which was expected based on their clinical indications, the most frequently assigned category was “cytochrome P-450 substrates” (*n* = 44) and “cytochrome P-450 enzyme inhibitors” (*n* = 33). Ion transporters such as organic anion transporters and p-glycoprotein also appeared frequently. In terms of chemical-based classes, the epigenetic compounds were most frequently “benzene and substituted derivatives” (Figure 3).

## 5. Functional Genomics of Epigenetically Active Drugs

Computational functional genomic analyses were performed based on targets retrieved from the HEMD and DrugBank databases as listed in Table 2. This was not corrected with further findings on PubMed. We did not detect a pattern of differences other than a temporal criterion for DrugBank entries. Therefore, it remained uncertain whether selective additions of drugs and especially only with their epigenetic targets would not be more likely to introduce bias, and furthermore, this would have made the analyses less reproducible than the clearly datable database-based information.

**Figure 4:**
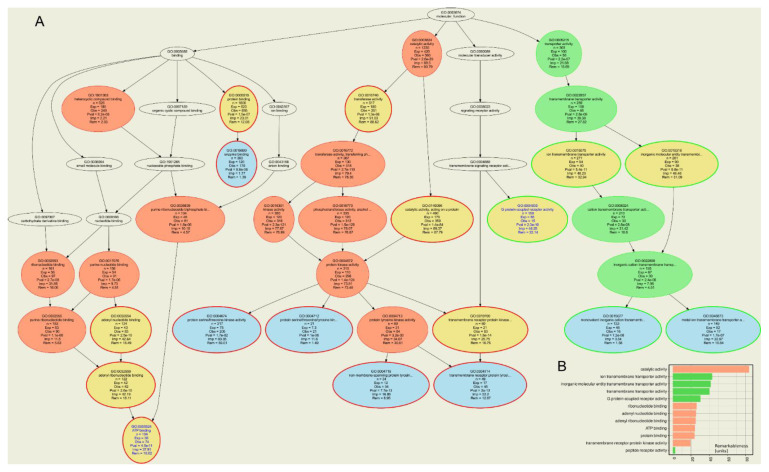
Computational functional genomics perspective on the molecular functions in which the genes encoding the targets annotated to the drugs that exert epigenetic effects. The figure displays the results of an overrepresentation analysis (ORA; *p*-value threshold, *t_p_* = 0.001 and Bonferroni α correction) of the *n* = 802 genes annotated to the drugs shown in Figure 2, of which *n* = 68 encode epigenetic enzymes. (**A**) Top-down representation of the annotations (GO terms) representing a systems biology perspective of the molecular functions modulated by the gene set. Each ellipse represents a GO term. The graphical representation follows the standard of the polyhierarchical organization of the GO knowledge base as a directed acyclic graph (DAG [114]). The color coding is as follows: no color: GO terms that are important for the DAG’s structure but do not have a significant *p*-value in Fisher’s exact tests. Red: significantly overrepresented nodes. Green: significantly underrepresented nodes. Blue: terms at the end (detail) of a branch of the DAG. In addition, the node’s text will be colored in blue to indicate that this node is a detail. Yellow: significant nodes with highest remarkableness in each path from a detail to the root, i.e., the so-called “headlines”. The margins of the ellipses indicate again overrepresentation (red) or underrepresentation (green). (**B**) Bar plot of the gene relevance in the functional genomics representation of the present gene set, quantified by remarkableness measure previously introduced [113]. The red bars indicate the most relevant overrepresented terms (headlines) and the green bars the most relevant underrepresented terms. The figure has been created using the R software package (v4.0.5 for Linux; https://CRAN.R-project.org/ [29]) and the R libraries “ABCanalysis” (https://cran.r-project.org/package=ABCanalysis [115]), “ggplot2” (https://cran.r-project.org/package=ggplot2 [36]) and “dbtORA” (https://github.com/IME-TMP-FFM/dbtORA [116]) with the DAG creation done with the GraphViz software package (https://graphviz.org [117]).

Approved or investigational drugs listed in the DrugBank database were identified to interact with a total of 2317 different human targets. The compounds identified to interact with 68 classical epigenetic enzymes interacted with 735 additional targets obtained as the set difference between all targets of these drugs and all epigenetic enzymes. ORA of all targets of the epigenetically active compounds versus all drug targets of approved or investigational drugs resulted in *n* = 320 GO terms significantly over- or underrepresented at the chosen α level, of which *n* = 42 belonged to the GO category “molecular processes”. The ORA results (Figure 4) showed that the drug targets that exerted effects on epigenetic enzymes were mainly involved in metabolic molecular functions such as “catalytic activity” (GO:0003824), “ribonucleotide binding” (GO:0032553), or “ATP binding” (GO:0005524) (Table 5). This was also the main result when only the targets belonging to the HEMD-based list of epigenetic enzymes were analyzed, with more specific terms such as “transcriptional regulator activity” indicating the narrower focus on epigenetic processes (Table 5). However, even when analyzing only the other targets of epigenetic agents, i.e., those not belonging to the HEMD-based list of epigenetic enzymes, metabolic and catalytic focus of molecular function prevailed. In contrast, molecular functions such as “G protein-coupled receptor activity” (GO:0004930) or “ion transmembrane transport” (GO:0034220) were underrepresented, i.e., fewer genes were annotated with these terms than expected in a random gene set (Figure 4). This is of particular interest given the epigenetic effects of ion channel blockers such as valproic acid or cocaine mentioned above or G protein-coupled receptor agonists such as opioids [19,20], suggesting that the epigenetic effects of these drugs are not systematic class effects but are likely due to particular chemical properties of the molecules.

**Table 5:** Main GO terms describing the molecular functions in which the genes encoding the targets annotated to the drugs that exert epigenetic effects.

Analysis	GO Term	Molecular Function	Observed Annotations	Expected Annotations	*p*-Value
**All targets**	GO:0003824	Catalytic activity	1230	424.9	2.61 × 10^−29^
	GO:0015075	Ion transmembrane transporter activity	271	93.6	5.36 × 10^−11^
	GO:0015318	Inorganic molecular entity transmembrane transporter activity	261	90.2	9.80 × 10^−11^
	GO:0022857	Transmembrane transporter activity	289	99.8	2.77 × 10^−9^
	GO:0004930	G protein-coupled receptor activity	159	54.9	2.29 × 10^−10^
	GO:0032553	Ribonucleotide binding	161	55.6	2.74 × 10^−8^
	GO:0030554	Adenyl nucleotide binding	124	42.8	2.53 × 10^−10^
	GO:0032559	Adenyl ribonucleotide binding	122	42.1	2.56 × 10^−10^
	GO:0005524	ATP binding	104	35.9	4.45 × 10^−11^
	GO:0005515	Protein binding	1806	623.9	1.49 × 10^−7^
	GO:0019199	Transmembrane receptor protein kinase activity	60	20.7	1.63 × 10^−14^
	GO:0001653	Peptide receptor activity	70	24.2	3.23 × 10^−4^
**Epigenetic targets**	GO:0008134	Transcription factor binding	101	3	2.46 × 10^−9^
	GO:0140110	Transcription regulator activity	100	2.9	2.08 × 10^−6^
	GO:0042826	Histone deacetylase binding	24	0.7	5.93 × 10^−7^
	GO:0003682	Chromatin binding	50	1.5	1.60 × 10^−4^
	GO:0048156	Tau protein binding	27	0.8	8.93 × 10^−48^
**Non-epigenetic targets**	GO:0016740	Transferase activity	517	163.4	2.61 × 10^−^
	GO:0140096	Catalytic activity, acting on a protein	490	154.9	2.35 × 10^−56^
	GO:0015075	Ion transmembrane transporter activity	271	85.6	8.22 × 10^−8^
	GO:0015318	Inorganic molecular entity transmembrane transporter activity	261	82.5	1.20 × 10^−7^
	GO:0032553	Ribonucleotide binding	161	50.9	1.96 × 10^−8^
	GO:0004930	G protein-coupled receptor activity	159	50.3	4.40 × 10^−8^
	GO:0032555	Purine ribonucleotide binding	153	48.4	1.11 × 10^−6^
	GO:0019199	Transmembrane receptor protein kinase activity	60	19	1.56 × 10^−16^
	GO:0017076	Purine nucleotide binding	156	49.3	1.47 × 10^−6^
	GO:0035639	Purine ribonucleoside triphosphate binding	134	42.3	3.05 × 10^−6^
	GO:0005515	Protein binding	1806	570.8	5.40 × 10^−5^

Results of an overrepresentation analysis (ORA; *p*-value threshold, *t_p_* = 0.001 and Bonferroni α correction) of the *n* = 802 genes annotated to the drugs shown in Figure 2, of which *n* = 68 encode epigenetic enzymes. The listed GO terms represent a functional genomics perspective on the molecular functions in which the analyzed compounds are involved. All selected terms (for the complete polyhierarchy; see Figure 4) qualify as headlines representing particular aspects (taxonomies) of the complete polyhierarchy at maximum coverage, certainty, information value and conciseness [113]. Three analyses were performed against all genetic targets annotated with approved or investigational drugs in the DrugBank database. We considered (i) all targets of the drugs queried in DrugBank with epigenetic effects, (ii) only their epigenetic targets, and (iii) only their non-epigenetic targets. Shown are the GO terms, the number of genes found annotated at each term, the expected number of genes in a random gene set, and the *p*-value of the deviation of the observation from this expectation.

## 6. Discussion

The dual line of information retrieval for epigenetic drug effects, based on PubMed literature search and DrugBank database query, yielded overlapping results, as expected. However, these were not completely redundant, highlighting the added value of this approach. For example, anandamide, an endocannabinoid, regulates skin differentiation though p38 and also p42/44 mitogen activated protein kinases [118]; however, anandamide is not a drug listed in the DrugBank and *MAPK* genes are not listed among primary epigenetic enzymes in the HEMD database. While the inclusion of anandamide may be in dispute, givinostat is certainly a drug, but in DrugBank its entry was commented as not yet fully annotated on 1 May 2021, and the targets were missing. The same was true for sulforaphane, while domatinostat was missing completely from the DrugBank database, as was chaetocin and some further hits from the literature search. This explains the only partial overlap between Table 3 and Table 4 on the one hand and Figure 2 on the other hand.

Interactions of drugs with epigenetic targets are comparatively rare among all interactions of approved or investigational drugs with human targets. That is, the 82 targets with which approved or investigational drugs interact account for 2.81% of the 2914 human drug targets listed in DrugBank. Of the 7213 unique agents listed as approved or investigational, the 122 drugs that interact with human epigenetic enzymes account for 1.53%, which is a significant underrepresentation of epigenetic drugs among all drugs in the selected groups (χ^2^ test [119]: χ^2^ = 18.264, df = 1, *p* = 1.923 × 10^5^). In addition, of the 31 drugs listed in DrugBank that have only epigenetic targets, which corresponds to the inclusion criterion in Table 3, 15 are annotated as “approved,” representing 0.2% of all drugs approved or under investigation listed in the DrugBank database.

Epigenetics in the pharmacologic context accounts only for a fraction of all research on epigenetics. A PubMed database query on 6 May 2021 for “(epigenetic* OR (DNA AND methyl*) OR (histone AND modific*) NOT review[PT])” using again the “RISmed” R package obtained 109,645 hits, i.e., the 3051 hits for the present overview account only for 2.78% of the total body of epigenetic publications. Moreover, only a few countries publish epigenetic research in the pharmacological context. However, the present bibliometric analyses showed a downward trend in publications and citations in the research field of pharmacological epigenetics. This may be related to the intense drug discovery activities in this field, which have led to a significant number of epigenetics therapeutics in the last decade.

The main clinical indications of the primary epigenetics drugs are oncological (Table 3). Other indications including immunological diseases such as rheumatoid arthritis [120] are emerging. Epigenetic drug discovery has mainly focused on the development of new compounds targeting enzymes involved in epigenetic regulation. The discovery of epigenetic effects of other drugs remained rare, as in the search for the mechanisms of action of valproate in persistent pain, of citalopram in psychiatric disorders, or in epigenetic opioid effects (Table 4). All of the latter effects are pleiotropic, i.e., effects off the primary targets of the drugs involved. The sparse knowledge of epigenetic effects of drugs contrasts somewhat with the knowledge of epigenetic effects of a variety of chemicals or even non-chemical influences, suggesting a more focused assessment of epigenetic effects of drugs would be worthwhile to address either desirable or side effects. However, the present computational functional genomic analyses (Table 5 and Figure 4) have shown that positive findings are more likely among drugs that interact with enzymes among their known targets, narrowing the focus of the search for pleiotropic epigenetic drug effects. This is supported by the frequency of drug categories that mention cytochrome P-450 or other enzymes or several of the known drug transporters (Figure 3). Of note, the search criterion in the DrugBank database was the epigenetic target of the drug and not the drug’s interaction with metabolizing enzymes or transporters, but nevertheless these categories emerged as typical of drugs interacting with epigenetic enzymes.

Epigenetic regulation can affect the pharmacokinetics of a drug via modulation of the expression of activating or inactivating enzymes or transmembrane transporters, with indirect consequences for the pharmacodynamic effects of the drug, or the pharmacodynamics of a drug directly via modulation of the expression of its targets. In fact, there is evidence that this plays a clinical role and contributes to pharmacological plasticity [121], which is considered a permanent deformation of the effects of a drug in a living system. Finally, it is in the ubiquity of epigenetic regulation of gene expression that epigenetic regulation can, in principle, affect any drug target and thus all drugs are subject to epigenetic influences via their targets. However, as with the epigenetic effects of drugs that are not primarily epigenetic, the effect of epigenetic regulation of targets as modulators of drug responses appears to have been rarely systematically studied. In most papers, epigenetic effects have merely been extrapolated from known regulations of target proteins as a likely case of pharmacological plasticity, but have not been directly shown to influence drug responses.

In addition to cancer therapy, epigenetically active drugs are expected to be relevant for a class of rare diseases that are directly related to epigenetics and are currently incurable. That is, imprinting disorders are a group of rare congenital disorders that affect growth, development, and metabolism and impact the quality of life of patients throughout their lives [122]. Genomic imprinting describes the monoallelic and parent-dependent expression of a subset of genes [123], meaning that gene expression is dependent on the origin of an allele from a particular parent including that only the maternal or paternal allele can be active. Expression of these gene sets is under coordinated epigenetic control, to which classical mechanisms of DNA methylation and histone modification contribute, e.g., via a specific methylation during gene expression. Genetic, epigenetic, or environmental insults that prevent imprinting from evading reprogramming can lead to imprinting disorders that affect growth, development, behavior, and metabolism [124] and even early embryonic failure and recurrent pregnancy loss when mutations develop in components of the human oocyte subcortical maternal complex (Demond, et al., 2019) [125]. There is increasing evidence that a subset of individuals affected by imprinting disorders have multi-locus imprinting disorders (MLID) [126], implying that these methylation defects in patients are not isolated events occurring at a specific disease-associated locus but that some of these patients may have imprinting disorders affecting additional imprinted regions present in multi-loci [127]. Several syndromes are associated with loss of methylation at specific imprinted loci. Among this group of pathologies known as imprinting disorders, Beckwith-Wiedemann syndrome is the most common congenital overgrowth disorder [128] and represents the paradigm of genetic imprinting disorders and cancer predisposition syndromes [129]. Research for therapies of imprinting disorders are still mainly in preclinical stages [130] such as the use of Histone Lysine Methyltransferase 2 for the treatment of the Prader-Willi syndrome, which is a complex and multisystem neurobehavioral disorder [131]. However, first human studies of the treatment of the Angelman syndrome have started in 2020 (https://clinicaltrials.gov/search/term=Angelman%20Syndrome). Repositioning of drugs is among strategies to find therapies for these rare diseases as it provides a faster and cheaper option to obtain active drugs than the development of novel compounds from scratch [132,133,134]. Several strategies have been proposed for the efficient search of drug repurposing candidates, of which those using a genomics-based approach are summarized in [116]. The ORA-based approach used in the fourth analysis step of the present report is among these proposals [135] and has been shown to provide promising results for rare diseases such as inherited syndromes with extreme pain phenotypes [136].

Finally, when considering further that different life events can affect a subject’s epigenetics; tailored therapies can be taken to a new level using a patient’s epigenome as the basis for precision medicine. However, epigenetic changes and especially non-chemical influences should be also viewed with caution, as enthusiastic reports of epigenetic changes, e.g., due to trauma, have also been criticized [137] and the claimed biological mechanism of transgenerationally transmitted trauma has been called implausible [138]. Indeed, many positive findings have not been replicated in independent studies, reminiscent of a similar situation a decade ago in genetic association studies, where enthusiastic positive findings often could not be replicated or, despite positive studies, the clinical benefit of the findings did not materialize, as in the case of pain [139]. Nevertheless, epigenetic transgenerational inheritance is an active research topic and several biological mechanisms have been proposed [140].

### Strengths and Limitations

In the present analyses, knowledge of epigenetic mechanisms was combined with knowledge of drug targets, their molecular biological functions, and bibliometric information queried from various databases. Bias may have been introduced by the selection of databases; however, the computational approach shifted the main possible bias to the content of the databases used. One hint at this was discussed above, where the classic literature search had yielded information that was not yet available in the DrugBank database. This is a common symptom of database-based analyses and must be considered when placing the information presented here in a broader context of the research area.

## 7. Methods

**Figure 5:**
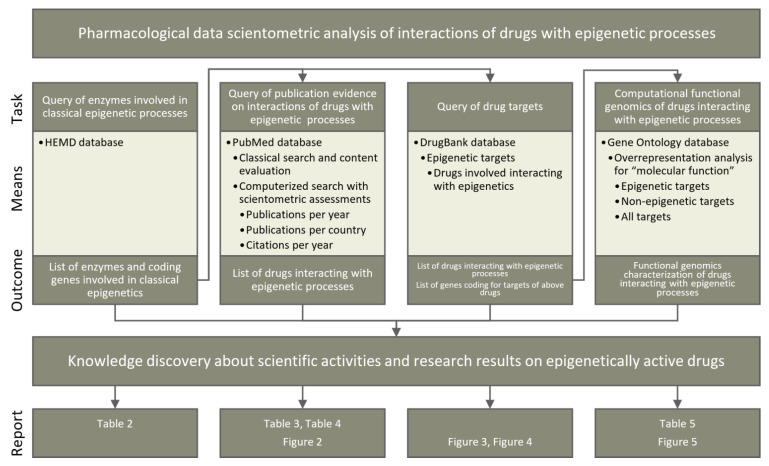
Schematic representation of the data analyses performed in four main steps. The main steps are shown as columns. The rows show (i) the main task pursued in each step, (ii) the main means by which each task was pursued, including the sources of information and methods used, (iii) the results obtained and outcomes from each step, including their possible use in subsequent analyses, and (iv) the bottom row indicates the table or figure in which the main results of each analysis step are presented.

Four lines of knowledge discovery for epigenetic implications of currently available drugs (Figure 5) were pursued in publicly available databases (Table 1). First, knowledge about enzymes involved in classical epigenetic processes was queried from the Human epigenetic enzyme and modulator database. Second, a classical literature search was performed among biomedical publications in the PubMed database. This was further investigated using bibliometric methods to assess the current scientific interest in epigenetics-related pharmacology. Third, pharmacological knowledge about drug targets was analyzed based on a query of the DrugBank database. Fourth, the functional genomic implications of the identified compounds and their targets were analyzed based on the GeneOntology database. All lines of knowledge discovery were computerized. The programming work was performed in the R language [141] using the R software package [29] (v4.0.5 for Linux), which is available free of charge in the Comprehensive R Archive Network at https://cran.r-project.org (accessed on 3 July 2021).

### 7.1. Query of Epigenetic Enzymes

Enzymes involved in the classical epigenetic reactions addressed in the present report were queried from the Human epigenetic enzyme and modulator database (HEMD) at http://mdl.shsmu.edu.cn/HEMD/ [26]. HEMD is an open access integrated tool of human epigenetic enzymes and chemical modulators for therapeutics. The data were downloaded in Extensible Markup Language (XML) format from http://mdl.shsmu.edu.cn/HEMDCommon/datasource/archive/HEMD_Release_040212_XF.tar.gz and the enzyme subclass, enzyme name, the Universal Protein Resource (UniProt) (https://www.uniprot.org [30]) ID and the Kyoto Encyclopedia of Genes and Genomes (KEGG) (https://www.genome.jp/kegg/ [28]) ID were retrieved from the individual XML files per enzyme using the R library “XML” (https://cran.r-project.org/package=XML [142]). The list was updated to current knowledge followed PubMed search for targeting potential new enzymes, and the National Center for Biotechnology Information (NCBI) accession numbers, gene names, and gene symbols were matched with the entries in the HUGO Gene Nomenclature Committee (https://www.genenames.org/ [27]) and UniProt databases (all accessed in April 2021). Subsequently, gene symbols and NCBI numbers were verified using the R library “org.Hs.eg.db” (https://bioconductor.org/packages/release/data/annotation/html/org.Hs.eg.db.html [143]).

### 7.2. PubMed Database Query

#### 7.2.1. Classical Publication Search on Epigenetic Drug Effects

Evidence for epigenetic effects of drugs with primarily non-epigenetic mechanisms of action was searched in the PubMed database at https://pubmed.ncbi.nlm.nih.gov/ (accessed on 4 May 2021). The search was performed in the classical way directly on the PubMed website and again using the R library “RISmed” (https://cran.r-project.org/package=RISmed [144]), using the search string “(epigen* AND (histon* OR chromatin* OR DNA) AND (modificat* OR alterat* OR modulat* OR changes OR changing) AND (methylase OR demethylase OR acethylase OR deacetylase OR ubiquinase OR deubiquinase OR phosphorylase OR dephosphorylase OR sumoylase) AND ((drug OR pharmaceut*) NOT (review[PT]))”. Only studies in which actual epigenetic changes occurred, i.e., DNA (de)methylation or histone modifications, were included in further analyses. Reports of changes in protein expression for which an epigenetic background was only a hypothesis were excluded, also papers mentioning epigenetic modulations but either not further addressing it or using animal research data or not showing clear evidence about epigenetic effects have been excluded from the analysis.

#### 7.2.2. Bibliometric Analyses of Publication Activities on Epigenetic Drug Effects

The results of the above PubMed database search were further evaluated for bibliometric analyses on scientific activities in the field of epigenetic effects of drugs. Specifically, the scientific output on epigenetic effects of drugs was analyzed descriptively as a function of publication year to capture the global evolution of scientific interest in this research topic. This was weighted by the total publication activity. The latter was determined by using an empty search string in the R-based PubMed database search, from which the number of publications per year by the hits found with the search string “review[PT]” was subtracted. In addition, the cumulative number of citations of each paper calculated from the publication date to the time of query was obtained using the “Cited” function of the “RISmed” R package. Cumulative references were averaged with respect the number of publications on epigenetics and drugs at the respective publication year. For comparability among scientific disciplines and topics, the mean number of expected publications was extrapolated using a mathematical model that takes into account both the time interval between publication date and literature query and the referencing behavior of the respective scientific community [32] as Equation (1):

(1) $$\langle {\bar{N}}_{cit}\rangle =\frac{{\bar{n}}_{cit,\Delta t}}{1-e^{-\frac{\Delta t}{\beta }}}$$

where $\langle {\bar{N}}_{cit}\rangle$ denotes the expected average number of references in total, ${\bar{n}}_{cit,\Delta t}$ is the average number of cumulative references calculated within the time span from the publication date until the query (Δt) and β denotes the time period in which a scientific article achieves 63 % of its total references, measured from the time of its publication. The weighting factor β depends on the citation behavior of the scientific community of the respective field. The search string used resulted mainly in publications from the fields of biochemistry and microbiology ($\beta_{bio}=5.4$) and pharmacology ($\beta_{pharma}=7.1$). For example, the only two papers from 1999 were cited 21 and 528 times since publication, respectively, which results in ${\bar{n}}_{cit,\;\Delta t}$ = 274 citations on average for epigenetics related publications from 1999 within a measurement period of Δt = 22 years. Based on the citation behavior of a readership dominated primarily by biochemists and molecular biologists ($\beta_{bio}$*)*, the model described above expects a total number of $\langle {\bar{N}}_{cit}\rangle$ = 279 citations.

Furthermore, the worldwide interest in this research topic was comparatively evaluated by visualizing the number of publications on a density-equalized cartogram [35]. The cartograms were calculated with a normalization of the number of publications on epigenetic drug effects to the population of the countries, averaged over the years in which publications on epigenetic drug effects were found. In addition, the visualization was performed after normalizing the number of publications to (i) the average population of the country from during the period of the publication date of the first hit of the present PubMed search to the last entry of the World’s population according to the United Nations Department of Economic and Social Affairs, Population Division (World Population Prospects 2019, Online Edition. Rev. 1, downloaded on 14 December 2020, from https://population.un.org/wpp/Download/Standard/Population/). In an additional analysis, the publications per country were normalized to the biomedical publication activity of the respective country, which was obtained by querying all non-review publications assigned to the respective country in PubMed with the search string “Country[i], [PL] NOT review[PT]”, where Country[i] denotes each country from which the papers found with the first search string originated. Only first author affiliation was considered, which may underestimate collaborative contributions for other countries, with the United States of America being both the country with the most publications and the top country for collaborative publications [145]. Since the results clearly indicated non-U.S. countries with high research activity, this simplification was considered acceptable for the present review purpose.

### 7.3. DrugBank Database Query

Comprehensive information about drugs and their molecular targets was queried from the DrugBank database [24] at https://go.drugbank.com (v5.1.8 dated 3 March 2021) (accessed on 22 April 2021). The database was downloaded as an XML file (https://go.drugbank.com/releases/5-1-8/downloads/all-full-database). The information contained in it was processed using the R package “dbparser” (https://cran.r-project.org/package=dbparser [146]). The drug targets were available encoded as UniProt IDs and converted into NCBI numbers of the coding genes using the R library “org.Hs.eg.db”. Additional information extracted from the DrugBank XML file included drug groups (e.g., approved, investigational, experimental, etc.), the organism from which individual targets were derived to limit analyses to human targets, and drug classes and categories to which the epigenetic drugs were assigned. The grouping of the DrugBank entries is based on the development status of the respective drug: Drugs that have been officially accepted for commercialization are grouped as “approved”, drugs that have reached clinical trials grouped as “investigational” and drugs that are researched pre-clinically are grouped as “experimental”. Subsequently, the drugs were matched with respect to their targets with the epigenetic enzymes identified in the HEMD database during the first step of the data analysis.

#### GeneOntology Knowledge Base Based Functional Genomics Analyses

Potential patterns among biological functions modulated by epigenetics-targeting drugs were investigated from a computational functional genomics perspective. Specifically, the biological roles of the genes coding for the targets of these drugs were identified based on the Gene Ontology (GO) knowledgebase [25] where the knowledge about genes is formulated using a controlled vocabulary of GO terms (categories), to which the genes [147] are annotated [148]. GO terms are related to each other by “is-a”, “part-of” and “regulates” relationships forming a polyhierarchy visualized as a directed acyclic (DAG [114]) also known as “knowledge representation” graph [25]. The GO database is searchable by three main categories, consisting of biological process, cellular component, and molecular function. The GO category of molecular function, defined as molecular-level activities performed by gene products that occur at the molecular level, such as “catalysis” or “transport” [25], was chosen for the description of the systemic drug actions.

Particular functions annotated to the targets of selected drugs were assessed by means of an over-representation analysis (ORA [149]). This calculated for each GO term whether among the genes annotated to it a subset of the drug target coding genes was more frequent than expected. The number of genes from the set of interests annotated with a particular GO term was compared to the number of genes if the set of genes were a random collection of genes. The significance of the difference was assessed by means of Fisher’s exact tests [150]. To restrict the results to the most important GO terms, the α level was set at 0.001 and corrected for multiple testing as proposed by Bonferroni [151], the minimum number of genes per GO term was set at *n* = 2, and only manually curated GO terms were used. Gene sets comprising (i) epigenetic targets of the drugs reviewed in this report, (ii) their non-epigenetic targets, and (iii) the union of both kinds of targets were analyzed against all targets of approved or invitational drugs listed in the DrugBank database. A more detailed description of the type of analyses performed can be found in [113]. These analyses were performed using our R library “dbtORA” (https://github.com/IME-TMP-FFM/dbtORA [116]), which in turn uses the data provided with the R packages “org.Hs.eg.db” (https://bioconductor.org/packages/release/data/annotation/html/org.Hs.eg.db.html [143]) and “GO.db” (https://bioconductor.org/packages/release/data/annotation/html/GO.db.html [152]) with the GeneOntology knowledge base version of 17 March 2021. As a basis for the selection of the most appropriate terms to describe the DAG, i.e., the terms that can serve as headlines for each branch of the DAG, the previously introduced remarkableness measure [113] was used.

## 8. Conclusions

Classical epigenetic molecular functions that target the regulation of DNA methylation or conformation and thus directly regulate its interaction with transcription factors as the initial step of gene expression are subject to modulation by drugs. This concerns a new class of drugs that have been developed in the last few decades with epigenetic targets in mind. In addition, this also concerns some classical drugs that were developed for non-epigenetic targets but for which evidence has been found that they also interact with epigenetic targets. The present analyses suggested that these drugs share an affinity for metabolic molecular processes but none of the typical other molecular drug functions such interaction with ion transport or G-protein-coupled receptors, making epigenetic pleiotropic effects an accidental drug property that probably relates to the specific chemical molecule structure rather than to a specific functional class. In addition to direct drug interactions with epigenetic targets, modulation of drug target expression or drug-metabolizing enzymes by epigenetic mechanisms is a general way of interaction between drugs and epigenetics; a few dedicated examples have been reported in the literature. The present data-driven analysis provided numerical information on drug interactions with epigenetic molecular functions. This includes the 68 enzymes catalyzing DNA methylation or histone modification reactions that interact with 122 different drugs. The study also revealed geographic differences in epigenetic drug research and suggested that research on epigenetic drug interactions is rare in both pharmacology and epigenetic research. Publication and citation activity have not increased in the past 5 years, which may be due to the successful development of a new class of epigenetic anticancer drugs before; however, the systematic search for epigenetic pleiotropic effects or on epigenetic pharmacological plasticity seems to have stalled despite the interest in epigenetic effects of many nonpharmacological chemicals.

## Figures and Tables

**Table 1:** Data sources and main FOSS tools. Publicly available data sources and freeware computational tools (FOSS, free and open source software) used to identify epigenetic drugs, their targets and to classify and visualize the biological functions of the latter (all accessed in April 2021).

Site Name	Uniform Resource Locator (URL)	Reference
AmiGO (search utility for GO)	http://amigo.geneontology.org/	[23]
DrugBank	https://go.drugbank.com	[24]
Gene Ontology (GO)	http://geneontology.org	[25]
Human epigenetic enzyme and modulator database (HEMD)	http://mdl.shsmu.edu.cn/HEMD/	[26]
HUGO Gene Nomenclature Committee	https://www.genenames.org	[27]
Kyoto Encyclopedia of Genes and Genomes (KEGG)	https://www.genome.jp/kegg/	[28]
NCBI gene index database	https://www.ncbi.nlm.nih.gov/gene/	
PubMed	https://pubmed.ncbi.nlm.nih.gov	
R software (v4.0.5)	https://CRAN.R-project.org/	[29]
Universal Protein Resource (UniProt) database	https://www.uniprot.org	[30]

**Table 2:** Epigenetic enzymes that interacted with approved or investigational drugs. Enzymes were queried from the human epigenetic enzymes and modulators database (HEMD) and selected on the basis of the entries in the DrugBank database.

Enzyme Action	Gene Name	Gene Symbol	NCBI Number
DNA methylation	DNA methyltransferase 1	*DNMT1*	1786
DNA methylation	DNA methyltransferase 3 alpha	*DNMT3A*	1788
DNA methylation	DNA methyltransferase 3 beta	*DNMT3B*	1789
DNA methylation	Trna aspartic acid methyltransferase 1	*TRDMT1*	1787
DNA demethylation	Alkb homolog 2, alpha-ketoglutarate dependent dioxygenase	*ALKBH2*	121642
DNA demethylation	Alkb homolog 3, alpha-ketoglutarate dependent dioxygenase	*ALKBH3*	221120
DNA demethylation	FTO alpha-ketoglutarate dependent dioxygenase	*FTO*	79068
DNA demethylation	Apolipoprotein B mrna editing enzyme catalytic subunit 1	*APOBEC1*	339
Histone methylation	Enhancer of zeste 1 polycomb repressive complex 2 subunit	*EZH1*	2145
Histone methylation	Enhancer of zeste 2 polycomb repressive complex 2 subunit	*EZH2*	2146
Histone demethylation	Lysine demethylase 5D	*KDM5D*	8284
Histone acetylation	Lysine acetyltransferase 2A	*KAT2A*	2648
Histone acetylation	Lysine acetyltransferase 2B	*KAT2B*	8850
Histone acetylation	Lysine acetyltransferase 5	*KAT5*	10524
Histone acetylation	Nuclear receptor coactivator 1	*NCOA1*	8648
Histone acetylation	Nuclear receptor coactivator 2	*NCOA2*	10499
Histone deacetylation	Histone deacetylase 1	*HDAC1*	3065
Histone deacetylation	Histone deacetylase 10	*HDAC10*	83933
Histone deacetylation	Histone deacetylase 2	*HDAC2*	3066
Histone deacetylation	Histone deacetylase 3	*HDAC3*	8841
Histone deacetylation	Histone deacetylase 4	*HDAC4*	9759
Histone deacetylation	Histone deacetylase 6	*HDAC6*	10013
Histone deacetylation	Histone deacetylase 8	*HDAC8*	55869
Histone deacetylation	Histone deacetylase 9	*HDAC9*	9734
Histone deacetylation	Sirtuin 1	*SIRT1*	23411
Histone deacetylation	Sirtuin 5	*SIRT5*	23408
Histone ubiquitination	MDM2 proto-oncogene	*MDM2*	4193
Histone ubiquitination	Ubiquitin like modifier activating enzyme 1	*UBA1*	7317
Histone deubiquitination	BRCA1/BRCA2-containing complex subunit 3	*BRCC3*	79184
Histone phosphorylation	Protein kinase AMP-activated catalytic subunit alpha 1	*PRKAA1*	5562
Histone phosphorylation	Protein kinase AMP-activated non-catalytic subunit beta 1	*PRKAB1*	5564
Histone phosphorylation	Cyclin dependent kinase 17	*CDK17*	5128
Histone phosphorylation	Cyclin dependent kinase 2	*CDK2*	1017
Histone phosphorylation	Cyclin dependent kinase 5	*CDK5*	1020
Histone phosphorylation	Cyclin dependent kinase 8	*CDK8*	1024
Histone phosphorylation	Death associated protein kinase 3	*DAPK3*	1613
Histone phosphorylation	Protein kinase, DNA-activated, catalytic subunit	*PRKDC*	5591
Histone phosphorylation	Glycogen synthase kinase 3 beta	*GSK3B*	2932
Histone phosphorylation	Component of inhibitor of nuclear factor kappa B kinase complex	*CHUK*	1147
Histone phosphorylation	LIM domain kinase 2	*LIMK2*	3985
Histone phosphorylation	Mitogen-activated protein kinase kinase kinase 12	*MAP3K12*	7786
Histone phosphorylation	Mitogen-activated protein kinase kinase kinase 20	*MAP3K20*	51776
Histone phosphorylation	Protein kinase C alpha	*PRKCA*	5578
Histone phosphorylation	Protein kinase C beta	*PRKCB*	5579
Histone phosphorylation	Ribosomal protein S6 kinase A3	*RPS6KA3*	6197
Histone phosphorylation	Ribosomal protein S6 kinase A4	*RPS6KA4*	8986
Histone phosphorylation	ATM serine/threonine kinase	*ATM*	472
Histone phosphorylation	Serine/threonine kinase 10	*STK10*	6793
Histone phosphorylation	Aurora kinase B	*AURKB*	9212
Histone phosphorylation	Aurora kinase C	*AURKC*	6795
Histone phosphorylation	Aurora kinase A	*AURKA*	6790
Histone phosphorylation	Checkpoint kinase 1	*CHEK1*	1111
Histone phosphorylation	Protein kinase N1	*PKN1*	5585
Histone phosphorylation	NIMA related kinase 9	*NEK9*	91754
Histone phosphorylation	P21 (RAC1) activated kinase 1	*PAK1*	5058
Histone phosphorylation	P21 (RAC1) activated kinase 2	*PAK2*	5062
Histone phosphorylation	Tousled like kinase 1	*TLK1*	9874
Histone phosphorylation	FYN proto-oncogene, Src family tyrosine kinase	*FYN*	2534
Histone phosphorylation	Janus kinase 2	*JAK2*	3717
Histone dephosphorylation	Protein phosphatase 2 catalytic subunit alpha	*PPP2CA*	5515
Histone dephosphorylation	Protein phosphatase 2 catalytic subunit beta	*PPP2CB*	5516
Histone dephosphorylation	Protein phosphatase 5 catalytic subunit	*PPP5C*	5536
Histone ADP-ribosylation	Poly(ADP-ribose) polymerase 1	*PARP1*	142
Histone ADP-ribosylation	Poly(ADP-ribose) polymerase 2	*PARP2*	10038
Histone ADP-ribosylation	Poly(ADP-ribose) polymerase family member 3	*PARP3*	10039
Histone de-ADP-ribosylation	*O*-glcnacase	*OGA*	10724
Histone citrullination	Peptidyl arginine deiminase 1	*PADI4*	29943
Histone biotinylation	Holocarboxylase synthetase	*HLCS*	3141

**Table 3:** Epigenetic therapeutics that have been developed with the purpose to exert epigenetic effects.

Substance	Main Target	Epigenetic Molecular Function Modified	Indications	References
**Abexinostat**	HDACs	Histone Acetylation Inhibitor	Non-Hodgkin’s and Hodgkin’s lymphoma	[38]
**Azacitidine**	DNMTs	DNA Methylation Inhibitor	Myeloid malignancies (FDA-approved)	[39,40]
**Belinostat**	HDACs	Histone Acetylation Inhibitor	Hepatocellular carcinoma (phase I/II)	[41]
**Chaetocin**	HMTs	Histone Methyltransferase inhibitor	Acute myeloid leukemia	[42]
**Chidamide**	HDI	Histone Deacetylase inhibitor	T cell lymphoma	[43]
**Decitabine**	DNMTs	DNA Hypomethylation Inhibitor	Myelodysplastic syndrome	[39]
**Domatinostat**	HDACs	Histone Acetylation Inhibitor	Various cancer	[44]
**Entinostat**	HDACs	Histone Acetylation Inhibitor	Solid tumors (phase I/II)	[45]
**Givinostat**	HDACs	Histone Acetylation Inhibitor	Haematological malignancies, Systemic-onset juvenile idiopathic arthritis (SOJIA)	[46,47]
**Guadecitabine**	HMTs	Histone Methyltransferase inhibitor	Various solid carcinomas and/or haematological malignancies	[48]
**Mocetinostat**	HDACs	Histone Acetylation Inhibitor	Various cancer	[49]
**Panobinostat**	HDACs	Histone Acetylation Inhibitor	Multiple myeloma	[50]
**Pinometostat**	HMTs	Histone Methyltransferase inhibitor	MLL-r leukemia patients	[51]
**Pracinostat**	HDACs	Histone Acetylation Inhibitor	Myelodysplastic syndrome	[52]
**Quisinostat**	HDACs	Histone Acetylation Inhibitor	Cutaneous T-Cell lymphoma	[53]
**Resminostat**	HDACs	Histone Acetylation Inhibitor	Solid tumors (phase I/II)	[54]
**Ricolinostat**	HDACs	Histone Acetylation Inhibitor	Multiple myeloma	[55]
**Romidepsin**	HDACs	Histone Acetylation Inhibitor	Advanced cutaneous T-cell lymphoma (CTCL) and peripheral T-cell lymphoma (FDA-approved)	[56]
**Seclidemstat**	LSD1	Lysine specific histone demethylase	Various cancer	[57]
**Sulforaphane**	HDACs, DNMTs	Histone Acetylation Inhibitor, DNA Methylation Inhibitor	Various cancer	[58]
**Tazemetostat**	HMTs	Histone Methyltransferase inhibitor	hematological malignancies and solid tumors	[59]
**Vorinostat**	HDACs	Histone Acetylation	Advanced cutaneous T-cell lymphoma (CTCL) (FDA-approved), multiple myeloma	[50,60]
**Zebularine**	DNMTs	DNA Methylation Inhibitor	Treatment of cancer cell lines	[61]

**Table 4:** Non-epigenetic drugs, i.e., drugs that have been discovered with other mechanisms of action but for which epigenetic effects have been successfully discovered after drug development.

Substance	Main Target	Epigenetic Consequences	Indications	References
**Cannabidol**	CB1/2 cannabinoid receptors	DNA hypermethylation	AIDS associated vasting syndrome, MS associated spastic symptoms, neuropathic pain	[62]
**Celecoxib**	Cyclooxycgenase 2	Reversal of the global DNA hypomethylation and the specific hypermethylation of the ER-α gene in rats with induced colon tumors	Inflammation, pain	[21]
**Cocaine**	Voltage-gated sodium channel	Decreased expression of histone methyltransferase G9a and subsequent lower methylation levels at H3K9	Local anesthetic	[63]
**Disulfiram**	Aldehyde dehydrogenase	DNA methyltransferase (DNMT) inhibitor	Prostate cancer	[64]
**Eflornithine (α-difluoromethylornithine)**	Ornithine decarboxylase	Reversal of the global DNA hypomethylation and the specific hypermethylation of the ER-α gene in rats with induced colon tumors	Facial hirsutism, sleeping sickness	[21]
**Escitaloprame**	Serotonin reuptake pump of neuronal membranes	Reduced mRNA expression for DNMTs and subsequent decreased gene-specific methylation levels	Major depression	[65]
**Fluoxetine**	Serotonin reuptake pump of neuronal membranes	Induction of methyl-CpG-binding proteins		[66,67]
**Gemcitabine**	DNA repair machinery	Inhibition of DNA repair process and the associated demethylation process à DNA hypermethylation	Cancer	[68]
**Hydralazine**	Ca^2+^ balance in the vascular smooth muscle	Hypomethylation by a stable interaction with DNMTs causing the inhibition of the methyltransferase activity	Hypertension, vasodilation	[69,70]
**Imatinib**	Tyrosine kinases abl, c-kit and PDGF-R	Increase in DNTM3A and EZ2H expression associated with promoter hypermethylation and down regulation of the tumor suppressor PTEN	Leukemia	[71]
**Opioids**	µ-opioid receptor	DNA hypermethylation	Pain, substitution therapy of opiate addiction	[20,72]
**Opioids**	µ-opioid receptor	Decreased expression of histone methyltransferase G9a and subsequent lower methylation levels at H3K9		[73]
**Tamoxifen**	Estrogen receptor	Down-regulation of estrogen receptor responsive genes pS2 and progesterone receptor due to promoter hypermethylation	Breast cancer	[74]
**Trichostatin A**	HDACs	Inhibitory effect upon histone deacetylase activity	Breast cancer	[75]
**Valproate**	Voltage gated sodium channel activity	Hyperacetylation of the N-terminal tails of histones H3 and H4	Epilepsy, bipolar disorder, diabetic peripheral neuropathy, hematological malignancies	[22]

## Data Availability

All data has been queried from publicly available sources that are referenced in the paper.
